# Management of Scapula Fractures at a Level 1 Trauma Centre in the United Kingdom

**DOI:** 10.7759/cureus.74947

**Published:** 2024-12-02

**Authors:** Basharat Ghafoor Khan, Muhammad Usman Ali, Sadia Farrukh, Muhammad Jamshed, Muhammad Umer Rasool, Kishan Gokaraju, Nick Aresti

**Affiliations:** 1 Trauma and Orthopaedics, Mersey and West Lancashire Teaching Hospitals NHS Trust, St. Helens, GBR; 2 Orthopaedics and Trauma, Health Education England North West London, London, GBR; 3 Biochemistry, Aga Khan University Hospital, Karachi, PAK; 4 Trauma and Orthopaedics, Hillingdon Hospital, London, GBR; 5 Trauma and Orthopaedics, North Manchester General Hospital, Manchester, GBR; 6 Trauma and Orthopaedics, Kings College Hospital NHS Foundation Trust, London, GBR; 7 Trauma and Orthopaedics, Barts Health NHS Trust, London, GBR

**Keywords:** demographics, fractures, management, nonoperative, operative, scapula

## Abstract

Background

Scapular fractures, an uncommon injury that can be brought on by a high-energy mechanism because of its proximity to the pectoral and shoulder muscles, are frequently linked to fatal injuries. This study aimed to compare surgical versus conservative treatment of scapular fractures and the results of treated patients.

Methods

The traumatic scapular fracture patients in this cross-sectional study (n = 391) were treated at a major trauma centre (level 1) in the United Kingdom between 2012 and 2018. Patients underwent a computed tomography (CT) scan of a scapula fracture with standard axial, coronal, and sagittal slices and a 3D reconstruction view based on an initial X-ray diagnosis. The Ideberg classification method was used to categorize all intra-articular scapular fractures. The two-tailed independent t-test was used to compare the surgical group to the traditionally conservative group. A p-value <0.05 was considered statistically significant.

Results

Scapular fractures were pre-dominant in males (82.09%, 321/391) with age (mean + SD) 43 + 1.23 years, and 76% (297/391) had 15 injury severity scores. Trauma with high energy was seen in 91% of cases for scapula fractures, whereas 93% were identified as polytrauma patients. The Advanced Trauma Life Support (ATLS) was performed in 92% of patients. The other injuries found were thorax (29.2%), limbs (21.2%), and spine (18.1%). A statistically insignificant (p-value = 0.135) difference was seen in the patients with surgery (EuroQol five-dimension five-level (EQ-5D-5L) score = 7.30) than nonoperative (EQ-5D-5L score = 6.14).

Conclusions

Based on our results, conservative management had better outcomes, but surgical options must be explored in selected cases as the difference in outcomes is not statistically significant.

## Introduction

The scapula joins the upper extremities to the axial skeleton and is a crucial component of the pectoral girdle. At the glenohumeral, acromioclavicular, and scapulothoracic joints, it connects to the humerus, clavicle, and thorax, respectively. Due to the scapula's unusual anatomy, fractures are extremely uncommon. The 18 muscles that arise from or insert into the scapula are responsible for coordinating the full range of motion in all three shoulder articulations [1]. These muscles work together to control six scapular movements: elevation, depression, upward/downward rotation, protraction, and retraction.

High-energy blunt force mechanisms are frequently responsible for causing scapula fractures [2,3]. All scapular fractures can be generated by direct force, but the impaction of the humeral head into the glenoid fossa might result in indirect force, which can cause glenoid and scapular neck fractures [4-7]. Most fractures are brought on by high-speed car accidents. Scapula fractures account for 3-5% of all shoulder injuries while only accounting for 0.4-1% of musculoskeletal injuries [8].

Even though there is a very low incidence of these fractures, they frequently are linked to an elevated “injury severity score" (ISS), and the patients are typically polytrauma patients who present with potentially fatal injuries [9].

Scapular fractures can be very painful and disrupt the normal functioning of the shoulder girdle due to issues such as malunion, nonunion, rotator cuff dysfunction, scapulothoracic dyskinesis, or impingement [10]. Scapula fractures can involve body, neck, glenoid, acromial, and coracoid fractures. Different classification systems are used along with the different treatment options based on different indications. Surgical indications usually involve open fractures, intra-articular fractures, floating shoulder, or massive angulations; however, these changes on a case-to-case basis. With the advancements in orthopaedic surgery today and a greater comprehension of how to treat injuries, such as in the shoulder girdle, there are a few accounts of the epidemiology and injury patterns of scapula fractures, but the current research is restricted to the epidemiology of the proximal humerus and clavicle [11]. Finding the research void in the treatment of scapular fractures is the study's justification.

To fill in this vacuum, a study was carried out to examine the medical files of scapula fracture patients who were admitted to a hospital between 2012 and 2018 to learn more about the prevalence and epidemiology of scapular fractures. The primary objective of this study is to compare the clinical outcomes of surgical versus conservative management of scapular fractures in patients treated at a level 1 trauma centre in the United Kingdom. The study aims to identify which treatment approach leads to better patient-reported outcomes and provide insights into the optimal management strategy for scapular fractures.

## Materials and methods

At Royal London Hospital, a major trauma centre in the United Kingdom, a cross-sectional study on patients (n = 391) with traumatic scapular fractures was carried out between 2012 and 2018. Ethics review project number 12887 was used to register this study with the Barts Health NHS Trust Clinical Governance Department. The closed or open scapula fractures were documented in the patients' medical records who were enrolled in the study. The demographics of patient age, gender, and body mass index (BMI) (kg/m^2^) were recorded, as well as the number of hospital visits and ICU admissions. Patients underwent conservative or surgical management.

In cases of polytrauma, related injuries were also reported, along with the anatomic site (intraarticular or extraarticular) and fracture personality (“open or closed fracture”). Before doing a computed tomography (CT) scan with the usual “axial, coronal, and sagittal” slices and a 3D reconstruction view, the scapula fracture was first diagnosed using X-rays. The following measurements were made: “glenopolar angle (GPA)," an articular step at the glenoid, and lateral base offset (LBO); however, angular deformation between fracture fragments and fracture fragment displacement was also noted. To classify all intra-articular scapular fractures, the Ideberg classification was considered [11]. Surgical management was offered to almost all the patients with open, comminuted, and severe intra-articular fractures.

The inclusion criteria included all the polytrauma patients with scapula fractures in ages >18 years old between 2012 and 2018 presented to major trauma centres. The exclusion criteria included age <18, stab injuries, and traumatic brain injuries. The EuroQol five-dimension five-level (EQ-5D-5L) was the primary outcome. The reliance on the EQ-5D-5L score as the primary outcome measure, without including other functional and clinical assessments, restricts the understanding of the true impact of surgical versus conservative management.

The EQ-5D-5L-based scoring system for treating scapular fractures principally emphasizes five crucial factors (mobility, self-care, exercise, discomfort, and anxiety) [12]. This was used as patient-reported outcomes to assess the functional status of patients in general. IBM SPSS Statistics for Windows, version 23.0 (released 2015, IBM Corp., Armonk, NY) was used to analyze the data. The surgical group and traditionally conservative group were compared using a two-tailed independent t-test with a statistically significant p-value <0.05.

## Results

**Figure 1:**
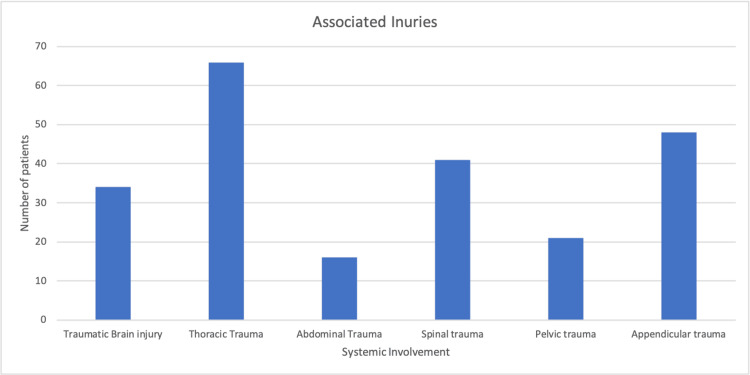
Different types of injuries in the patients. The chart above gives the number of injuries associated with patients with scapula fractures. As demonstrated from the data above, the most commonly associated injuries with scapula fractures are thoracic injuries, followed by appendicular injuries.

**Figure 2:**
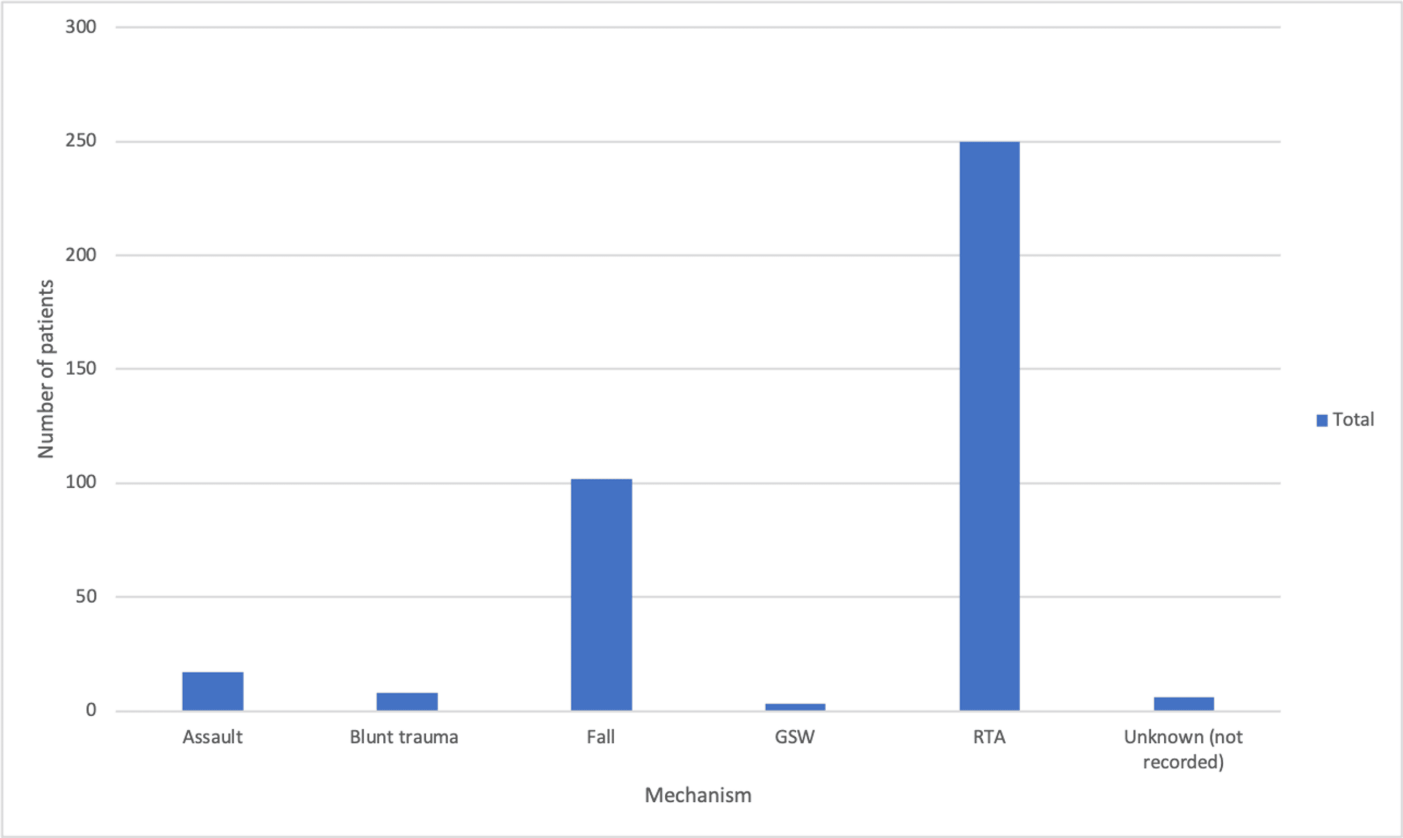
Pattern of injury The most common mechanism of injury seen in patients was road traffic accidents (RTAs), followed by falls, which demonstrate that high-energy injuries and other injuries should be looked into with some exceptions.

A total of n = 391 scapula fractures were treated, and it was found that age (mean +/- standard deviation (SD)) of adults (369/391) was 45 +/- 17.9 years with males (321/391) (mean +SD) 43+/-1.23 and 21 (5.3%) had bilateral scapular fractures. Many cases (91%) were of high-energy trauma and responsible for scapula fractures, and 93% were identified as polytrauma patients. When the ISS was observed, 297 (76%) had a score greater than 15. The other injuries were also monitored, and it was found thorax trauma was followed by limbs and spine traum, as demonstrated in Figure 1. As far as the mechanism of injuries is concerned, road traffic accidents (RTAs) were the most common mechanism, followed by falls, as demonstrated in Figure 2. 

Some patients (29%, 115/391) had hemodynamic instability with a 95% confidence interval (CI) of (24.8%, 33.8%), 11% had neurological instability with a 95% CI of (7.9%, 14.1%), and 0.76% of the patients were transported to the hospital dead with 95% CI of (0%, 1.56%). Ninety-six per cent of the patients underwent CT scans; of these, 32% later requested a CT shoulder/scapula, and 10% were detected by 3D CT reconstruction technology. Due to the tight muscle wrapping around the scapula, 25% of fractures had an extra-articular pattern, whereas 75% of fractures had an intra-articular pattern, as shown in Table 1. 

**Table 1:** Types and frequency of scapula fractures in adults

Type of fracture pattern (n = 369)	Number of fracture (n = 369)	Joint involvement	n(%)
Glenoid	51 (14)	Intraarticular	51 (14)
Neck	31 (8.4)	Intraarticular	18 (5)
		Extraarticular	13 (3.5)
Body	173 (47)	Intraarticular	173 (47)
Coracoid	38 (10)	Intraarticular	16 (4.3)
		Extraarticular	22 (6.5)
Acromion	35 (9)	Intraarticular	13 (3.5)
		Extraarticular	25 (7)
Clavicle	38 (10)	Intraarticular	5 (1.3)
		Extraarticular	30 (8)
Acromioclavicular joint	3 (1)	Intraarticular	1 (0.2)
		Extraarticular	2 (0.5)

According to the Ideberg classification, the most common glenoid fracture pattern was 27 scapula fractures labelled as grade V, followed by scapula fractures 11 and 5 categorized as grades III and II, respectively. Grade IV fractures were seen in three patients followed by 2 in both types I and VI. Most commonly, surgically managed injuries were grade V, followed by grade III, as shown in Table 2.

**Table 2:** Ideberg classification of scapula fractures

Ideberg classification	Number of scapula fractures	Managed surgically
IA	2	0
IB	0	0
II	5	1
III	11	4
IV	3	0
V	27	14
VI	2	1

Only 48 patients in total were operated on with 26 extraarticular and 22 intraarticular fractures. Only 90 patients had their injuries followed up, and of those, 83 had "good union rates," compared to five who had "nonunions," and only one had a reverse total shoulder arthroplasty.

Fifty-seven patients responded to the EQ-5D-5L survey asking about their quality of life. Out of these, 44 patients received conservative care, while 13 patients underwent "open reduction internal fixation" (ORIF). The mean EQ-5D-5L for patients treated non-operatively was 6.14+/- 2.17 with a p-value of 0.135, which was not statistically significant. The mean EQ-5D-5L for patients treated surgically was 7.30 +/- 4.20. Table 3 shows individual EQ-5D-5L responses for surgical patients. 

**Table 3:** EQ-5D-5L score for the surgical patients EuroQol five-dimension five-level (EQ-5D-5L) scores for the patients who underwent surgery are elaborated in this table, and the sum of these was compared with those of the non-surgical treatment. A P-value of <0.05 was used as statistically significant and was used for the calculation and used in this study.

Sex	Age	Mechanism	Mobility	Self-care	Activity	Pain	Anxiety
M	38	RTA	1	1	1	1	1
F	51	Fall	3	1	2	3	1
F	71	RTA	1	1	1	2	1
M	51	RTA	1	1	1	1	1
M	32	RTA	4	4	4	4	2
M	44	RTA	1	1	1	2	2
M	63	RTA	3	3	3	2	2
M	20	RTA	1	1	1	2	1
M	42	RTA	1	1	1	1	1
M	25	RTA	1	1	1	1	1
M	35	RTA	1	1	1	1	1
M	26	RTA	1	1	1	1	1
M	40	RTA	1	1	1	1	1

Although there is no statistically significant difference in both studies, given the low number of patients in the surgical group, these results are underpowered, so non-significant results may be due to the insufficient sample size rather than the absence of a true effect.

## Discussion

To the best of our knowledge, an insignificant difference between operative versus nonoperative management was found, but the non-operative group scored better on the EQ-5D-5L test than the operative group. However, the small sample size is a limiting factor in developing a definite conclusion on which treatment will be better in scapular injuries. The investigation was carried out in a tertiary care referral facility and a major trauma centre, which is a regional centre for polytrauma cases. It was discovered that between 2011 and 2019, there was a spike in scapula fractures and polytrauma patients. Tatro et al. mentioned a survey from the US National Trauma Data Bank that revealed that patients (106,119) had scapula fractures between 2002 and 2012 [3]. Other studies [4,13,14] cited similar findings. The use of CT scans as a diagnostic tool for trauma sufferers to determine the prevalence of scapula fractures has increased recently [3].

The age distribution is the main factor in defining damage, as the proportion of pedestrians suffering from a scapula fracture decreases with age up to 37. However, it was discovered that the most common fractures were caused by motor vehicle accidents in both genders among patients under 20 and >20, followed by falls from height (Figure 2).

Ninety percent of simultaneous injuries were caused by the mechanism of injury in this investigation, whereas the remaining injuries were isolated [3]. This study's findings that high-energy trauma produces an increase in the incidence of scapular fractures, which appear to be related to an increase in concurrent injuries. A retrospective analysis of 113 patients with scapula fractures revealed that 96% of them also had other injuries, the most frequent of which were chest injuries (ribs 37%, hemopneumothorax 29%, and pulmonary contusion 8%). In addition, 34% of cases involved brain injuries, 25% of patients had clavicle fractures, 3.5% had brachial plexus injuries, and 1% had subclavian artery injuries [5].

In another study conducted by Brown et al., it was discovered that 40.2% of patients had concurrent pneumothorax, 18% had hemothorax, and 18% had clavicle fractures [14]. Scapular fractures are high-energy fractures, and trauma patients without scapular fractures [9], including upper extremities, thoracic, and pelvic ring injuries, accounting for 80-95% of cases [3,4,13,14]. Pneumothorax, pulmonary contusion, vascular damage, splenic or hepatic lacerations, and closed head injury are among the various life-threatening events with a 15% death rate [2,7].

In addition, radiography is used as a major diagnostic tool, especially in polytrauma and unconscious patients [15,16]. Since there are multiple muscles that protect the scapula, a severe impact injury is necessary for scapula fractures. As a result, a rise in the mean ISS of 26 primarily in polytrauma patients can be used to explain an increase in RTAs and scapula fractures. Therefore, scapula fractures have a 10-15% death rate associated with "pulmonary sepsis or traumatic brain injuries" [9].

According to Launonen et al., it was found that simultaneous injuries occurred in 55% of patients. The most common injury found was blunt chest trauma (23.4%), followed by clavicle fracture (16.5%) and cervical spine or head injury (6.9%) [4]. Due to higher injury severity ratings and morbidity increases, the care options are restricted in patients with scapular fractures. The management strategies are constrained by related injuries since individuals with scapular fractures have greater injury severity ratings and increased morbidity. According to our research, 66% of the patients had thoracic trauma, compared to 48% for other orthopaedic injuries, 34% for head injuries, 3% for brachial plexus injuries, and 41% for spine injuries. The body of published research and our findings are in agreement.

Nordqvist and Peterson et al. examined 37 nonoperatively treated glenoid neck fractures with displaced position, after a 10- to 20-year follow-up, and discovered that functional outcomes were fair or poor in 32% of cases [17]. These conclusions were reached after a review of 37 nonoperatively treated fractures. Similar findings were made by Ada and Millar, who noted that of the 16 patients in their series who received conservative treatment for displaced scapular fractures of the neck; 50% of them felt nighttime pain, 40% had weakness with abduction, and 20% had reduced range of motion. These results were based on those of the 113 patients in the Ada and Millar case series [5].

Hardegger et al. [18] claimed that displaced glenoid neck fractures alter how the glenohumeral joint connects to the acromion and nearby muscle origins, which ultimately leads to a functional imbalance [19-22].

In a systematic analysis, Zlodowski et al. found that 77% of patients who underwent nonoperative treatment for scapular neck fractures without glenoid involvement experienced excellent or satisfactory results [23], which can be compared with limited data as per this study, showing better outcomes with non-surgical management.

Around 50% of all scapular fractures, according to research, include the scapular body and spine [24]. The healing of these fractures is typically aided by non-operative therapy, such as casting and bracing [2]. Conservative therapy for scapular body fractures has been shown to have favourable outcomes, including fracture union and good functional results [2,6,25].

In addition, there are no publicly available comparison studies that show that surgical intervention produces better results. For certain individuals, surgery will undoubtedly improve the outcomes, and the management process for the surgeons will be influenced by an evaluation of all the information available on the pattern of fracture and the patient characteristics. Although they can be used as a strong basis for decision-making and as a guide for decision-making, GPA distortion, medialization, and scapula angulation cannot be used as a clear indication for surgery [26].

In this study, the EQ-5D-5L showed no statistically significant difference (p-value = 0.135) as discussed in the results in the limited number of patients. However, compared to patients who received conservative treatment, individuals who underwent surgery for scapula fractures were four times less common. It is crucial to understand that surgical intervention is required more commonly for intra-articular glenoid fractures than for extra-articular scapula fractures. This finding might have a big impact on the direction of future research.

The results of this study contribute to the growing body of evidence demonstrating that scapula fractures occur more frequently than previously thought. Our study does, however, contain certain restrictions. In the first place, the data supplied are from a single institute, as opposed to the trauma database Trauma Audit and Research Network (TARN), which can provide a more complete picture of these injuries and far more persuasive evidence. The study's second limitation is the unknown number of patients who were only seen during accident and emergency (A&E) attendance with no regular follow-ups in the fracture clinic. In addition, there was no statistically meaningful change seen in the small number of patients whom we were able to contact and assess their EQ-5D-5L ratings. By contrast, there were four times fewer patients who needed surgery for scapula fractures than those who were treated conservatively. This might introduce prejudice for non-surgical management and lead to biased interpretation.

Moreover, there were no patient-reported outcome measures or clinical outcomes that were extensively compared across the operative and non-operated groups; it may not be concluded if a good clinical outcome corresponds with a high quality of life on the EQ-5D-5L.

## Conclusions

The study found no statistically significant difference in EQ-5D-5L scores between patients managed conservatively and those treated surgically. These results suggest that conservative management can be an effective option for many patients. However, the role of surgery in specific cases, especially those with complex fractures, warrants further investigation.
