# Correction: Management of Scapula Fractures at a Level 1 Trauma Centre in the United Kingdom

**DOI:** 10.7759/cureus.c382

**Published:** 2025-11-20

**Authors:** Basharat Ghafoor Khan, Muhammad Usman Ali, Sadia Farrukh, Muhammad Jamshed, Muhammad Umer Rasool, Kishan Gokaraju, Nick Aresti

**Affiliations:** 1 Trauma and Orthopaedics, Mersey and West Lancashire Teaching Hospitals NHS Trust, St. Helens, GBR; 2 Orthopaedics and Trauma, Health Education England North West London, London, GBR; 3 Biochemistry, Aga Khan University Hospital, Karachi, PAK; 4 Trauma and Orthopaedics, Hillingdon Hospital, London, GBR; 5 Trauma and Orthopaedics, North Manchester General Hospital, Manchester, GBR; 6 Bone Tumour Unit, Royal National Orthopaedic Hospital, London, GBR; 7 Trauma and Orthopaedics, Barts Health NHS Trust, London, GBR

This article has been corrected to include Kishan Gokaraju as an author after he was mistakenly omitted during article submission. All authors have agreed to this addition.

